# Chronic administration of aripiprazole activates GSK3β-dependent signalling pathways, and up-regulates GABA_A_ receptor expression and CREB1 activity in rats

**DOI:** 10.1038/srep30040

**Published:** 2016-07-20

**Authors:** Bo Pan, Xu-Feng Huang, Chao Deng

**Affiliations:** 1 Antipsychotic Research Laboratory, Illawarra Health and Medical Research Institute, Wollongong, 2522, NSW, Australia; 2 Centre for Translational Neuroscience, School of Medicine, University of Wollongong, Wollongong, 2522, NSW, Australia

## Abstract

Aripiprazole is a D_2_-like receptor (D_2_R) partial agonist with a favourable clinical profile. Previous investigations indicated that acute and short-term administration of aripiprazole had effects on PKA activity, GSK3β-dependent pathways, GABA_A_ receptors, NMDA receptor and CREB1 in the brain. Since antipsychotics are used chronically in clinics, the present study investigated the long-term effects of chronic oral aripiprazole treatment on these cellular signalling pathways, in comparison with haloperidol (a D_2_R antagonist) and bifeprunox (a potent D_2_R partial agonist). We found that the Akt-GSK3β pathway was activated by aripiprazole and bifeprunox in the prefrontal cortex; NMDA NR2A levels were reduced by aripiprazole and haloperidol. In the nucleus accumbens, all three drugs increased Akt-GSK3β signalling; in addition, both aripiprazole and haloperidol, but not bifeprunox, increased the expression of Dvl-3, β-catenin and GABA_A_ receptors, NMDA receptor subunits, as well as CREB1 phosphorylation levels. The results suggest that chronic oral administration of aripiprazole affects schizophrenia-related cellular signalling pathways and markers (including Akt-GSK3β signalling, Dvl-GSK3β-β-catenin signalling, GABA_A_ receptor, NMDA receptor and CREB1) in a brain-region-dependent manner; the selective effects of aripiprazole on these signalling pathways might be associated with its unique clinical effects.

Aripiprazole is a unique antipsychotic drug with a pharmacological profile different from other available antipsychotics, and this difference has been attributed to its partial agonism for the dopamine D_2_ receptor (D_2_R). A large body of evidence has shown that most antipsychotic drugs (including aripiprazole and haloperidol) have a potent affinity at the D_2_Rs^1^, regulating the D_2_R downstream protein kinase B (Akt)-glycogen synthase kinase 3 beta (GSK3β) and protein kinase A (PKA) signalling pathways^2^. In addition, these two signalling pathways are also linked to several other pathways or substrates, such as the dishevelled(Dvl)-GSK3β-β-catenin pathway, γ-aminobutyric acid (GABA)_A_ receptor and cAMP-responsive element-binding protein 1 (CREB1)^3^,^4^,^5^.

GSK3β-dependent signalling pathways are involved in the pathophysiology of schizophrenia and the actions of antipsychotics^4^. First, GSK3β is a major downstream regulator of D_2_-like receptors that is targeted by most antipsychotics^6^. It has been reported that chronic haloperidol treatment phosphorylates GSK3β, and inhibits its activity, which is associated with increased phosphorylation levels of Akt in the frontal cortex^7^. Second, antipsychotic administration can also influence the Dvl-GSK3β-β-catenin signalling pathway. Several studies have reported that various antipsychotics (including clozapine, haloperidol, risperidone, olanzapine and aripiprazole) were able to increase phosphorylation of GSK3β and expression of Dvl and β-catenin in the frontal cortex and striatum^8^,^9^,^10^,^11^,^12^.

The G protein-dependent PKA pathway is another downstream signalling pathway of D_2_-like receptors. PKA signalling has been shown to be related to the pathophysiology of schizophrenia by a post-mortem study^13^. An *in vivo* study has indicated that acute administration of haloperidol and olanzapine increased the expression of PKA catalytic subunits in the rat caudate putamen (CPu)^14^; PKA signalling has also been elevated by acute administration of haloperidol in the striatum^15^; and furthermore, the activity of the PKA pathway and expression of PKA regulatory subunits in the striatum were elevated after a 3-week administration of haloperidol in various brain regions, but decreased by clozapine administration^16^. A recent study has shown that 1-week administration of aripiprazole increased PKA phosphorylation in the nucleus accumbens (NAc), but reduced it in the CPu, while haloperidol decreased it in both the NAc and CPu^17^.

The GABA_A_ receptor has been reported to be involved in the pathophysiology of schizophrenia^18^. Increased binding density of GABA_A_ receptors have been found in the prefrontal cortex (PFC)^19^,^20^, cingulate cortex^21^, superior temporal gyrus^22^ and hippocampus^23^ of schizophrenic subjects. Antipsychotic administration has been shown to have various effects on GABA_A_ receptors. It has been reported that 1-week treatment with both haloperidol and olanzapine increased the binding density of [^3^H]-Muscimol labelled GABA_A_ receptors^24^ in the PFC. Zink and colleagues^25^ have found that haloperidol administration for 6 months increased the binding density of GABA_A_ receptors in the CPu and core of the NAc, but reduced it in the PFC, anterior cingulate and infralimbic cortex; and 6-month clozapine administration reduced the bindings of GABA_A_ receptors in the anterior cingulate and infralimbic cortex. Recent data suggested that expression of GABA_A_ receptors in the rat NAc was elevated by 1-week aripiprazole administration, probably by activation of the PKA pathway^17^.

CREB1 is also a downstream substrate of the PKA pathway^26^. Novel variants in the *CREB1* gene have been identified in schizophrenic subjects, and a relationship between CREB1 and the positive symptoms of schizophrenia has been proposed^27^. Previous *in vivo* and *in vitro* studies have shown that haloperidol increased phosphorylation levels of CREB1^28^,^29^,^30^. Additionally, amisulpride, clozapine and olanzapine also elevated phosphorylation levels of CREB1 *in vitro*^31^,^32^,^33^. Furthermore, a 3-week injection of aripiprazole changed the phosphorylation levels of CREB1 in the PFC and striatum of rats, probably through NMDA receptors^34^. A recent short-term study has reported that aripiprazole increased the gene and protein expression of CREB1, probably through the PKA pathway, in the NAc of rats^17^.

N-methyl-D-aspartate receptors (NMDARs) have been shown to be associated with schizophrenia and can be modulated by antipsychotics^5^. Blockade of NMDARs exacerbates symptoms in schizophrenia individuals^35^ or induces abnormal behaviours that resemble the symptoms and cognitive deficits of schizophrenia in healthy subjects^36^,^37^. Previous studies showed that antipsychotic drug administration had various effects on NMDARs, depending on the classes of antipsychotics, treatment methods (e.g. dosages, modes of drug delivery and time frames) and brain regions^38^,^39^,^40^,^41^. Therefore, the NMDAR subunits were also examined in the present long-term study.

Recent *in vivo* studies showed that acute and short-term administration of aripiprazole – a potent D_2_R partial agonist – displayed different effects that cannot be achieved by haloperidol (a typical antipsychotic and a potent D_2_R antagonist) and bifeprunox (a potent D_2_R partial agonist), providing preliminary *in vivo* evidence that neither D_2_R partial agonism nor D_2_R antagonism could be solely explain the pharmacological mechanism and unique clinical effects of aripiprazole^17^,^42^. It should also be noted that in clinics, antipsychotics require a long treatment period to reach maximum therapeutic effect, and thus are often used chronically^43^. All previous chronic studies administered antipsychotics by various methods (e.g. mixing in drinking water, daily injection), rather than the oral administration that mimics the clinical situation. The chronic effects of aripiprazole by such oral administration are not clear. Therefore, the present study investigated the effects of 10-week oral administration of aripiprazole by examining PKA signalling, Akt-GSK3β and Dvl-GSK3β-β-catenin pathways, GABA_A_ receptors and CREB1 activity, in comparison with haloperidol and bifeprunox.

## Results

### The effect of antipsychotics on Akt and GSK3β activity

#### PFC

**Figure 1:**
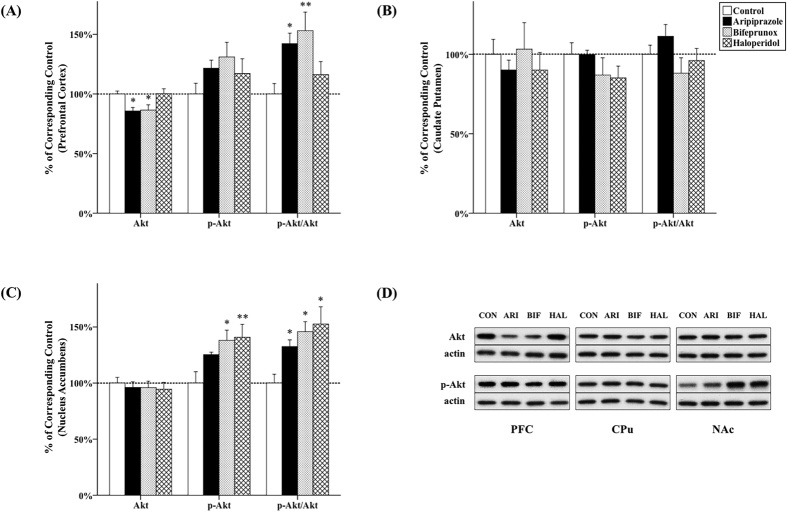
Effects of three antipsychotics on Akt activity. The effects of aripiprazole (ARI), bifeprunox (BIF) and haloperidol (HAL) on Akt activity were measured in the prefrontal cortex (**A**), caudate putamen (**B**) and nucleus accumbens (**C**). The representative bands of Western blot are shown in (**D**). Akt was quantified at 60 kDa; p-Akt was quantified at 60 kDa. The data were normalised by taking the average value of the control group as 100% and expressed as mean ± S.E.M. (*p ≤ 0.05, **p < 0.01 *vs* the control).

**Figure 2:**
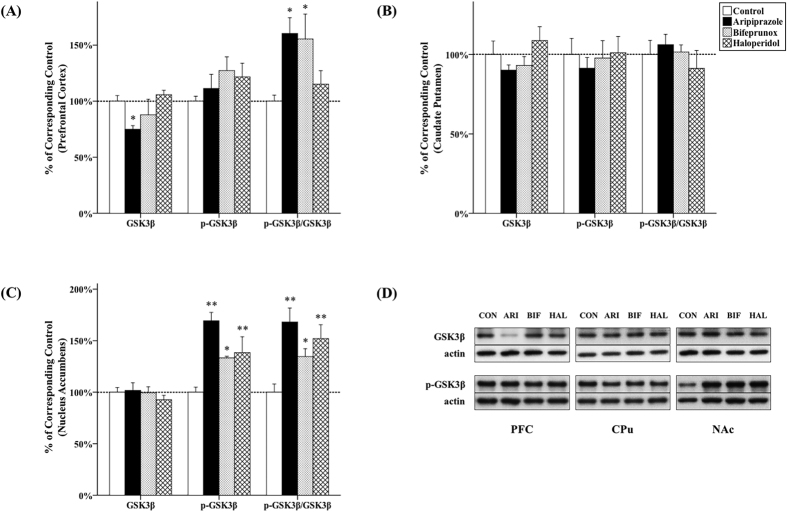
Effects of three antipsychotics on GSK3β activity. The effects of aripiprazole (ARI), bifeprunox (BIF) and haloperidol (HAL) on GSK3β activity were measured in the prefrontal cortex (**A**), caudate putamen (**B**) and nucleus accumbens (**C**). The representative bands of Western blot are shown in (**D**). GSK3β was quantified at 46 kDa; p-GSK3β was quantified at 46 kDa. The data were normalised by taking the average value of the control group as 100% and expressed as mean ± S.E.M. (*p ≤ 0.05, **p < 0.01 *vs* the control).

It has been shown that the expression of total Akt and total GSK3β was significantly affected by 10-week antipsychotic drug administration in the PFC (Akt, *F*_3, 20_ = 5.201, *p* = 0.004; GSK3β, *F*_3, 20_ = 3.083, *p* = 0.026); however, the levels of p-Akt and p-GSK3β were not significantly affected (p-Akt, *F*_3, 20_ = 1.554, *p* = 0.232; p-GSK3β, *F*_3, 20_ = 1.208, *p* = 0.332). The ratio of p-Akt/Akt (*F*_3, 20_ = 4.523, *p* = 0.007) and p-GSK3β/GSK3β (*F*_3, 20_ = 4.112, *p* = 0.010) was also significantly affected by antipsychotic drug administration. Post-hoc testing demonstrated that chronic administration of both aripiprazole and bifeprunox reduced expression of Akt (aripiprazole, −14.2%, *p* = 0.014; bifeprunox, −13.6%, *p* = 0.008) in the PFC (Fig. 1A,D). Additionally, administration of aripiprazole significantly suppressed GSK3β expression (by 25.0%; *p* = 0.043) compared with controls (Fig. 2A,D). The levels of p-Akt and p-GSK3β were not significantly affected. In addition, aripiprazole and bifeprunox administration significantly increased the ratio of p-Akt/Akt (aripiprazole, *p* = 0.021; bifeprunox, *p* = 0.005) (Fig. 1A). The ratio of p-GSK3β/GSK3β was also increased by administration of aripiprazole (*p* = 0.012) and bifeprunox (*p* = 0.019) (Fig. 2A).

#### CPu

Chronic antipsychotic drug administration had no significant effects on the levels of Akt, p-Akt (Fig. 1B,D), GSK3β and p-GSK3β (Fig. 2B,D) in the CPu compared with controls (all *p* > 0.05).

#### NAc

Drug treatment was able to significantly change the levels of p-Akt (*F*_3, 20_ = 4.315, *p* = 0.009), p-GSK3β (*F*_3, 20_ = 9.798, *p* < 0.001), as well as the ratio of p-Akt/Akt (*F*_3, 20_ = 5.268, *p* = 0.004) and p-GSK3β/GSK3β (*F*_3, 20_ = 7.024, *p* = 0.001) in the NAc. Compared with the control group, both bifeprunox and haloperidol administration significantly elevated the levels of p-Akt in the NAc (bifeprunox, +38.1%, *p* = 0.010; haloperidol, +40.8%, *p* = 0.006); aripiprazole also tended to increase the levels of p-Akt (+25.3%, *p* = 0.072) (Fig. 1C,D). All three drugs increased the ratio of p-Akt/Akt (aripiprazole, *p* = 0.044; bifeprunox, *p* = 0.007; haloperidol, *p* = 0.002) (Fig. 1C). Furthermore, administration of all three was able to elevate the levels of p-GSK3β (aripiprazole, +69.3%, *p* < 0.001; bifeprunox, +33.2%, *p* = 0.026; haloperidol, +38.4%, *p* = 0.010) (Fig. 2C,D) and significantly increased the ratio of p-GSK3β/GSK3β (aripiprazole, *p* = 0.001; bifeprunox, *p* = 0.048; haloperidol, *p* = 0.005) (Fig. 2C).

### The effect of antipsychotics on Dvl-3 and β-catenin expression

**Figure 3:**
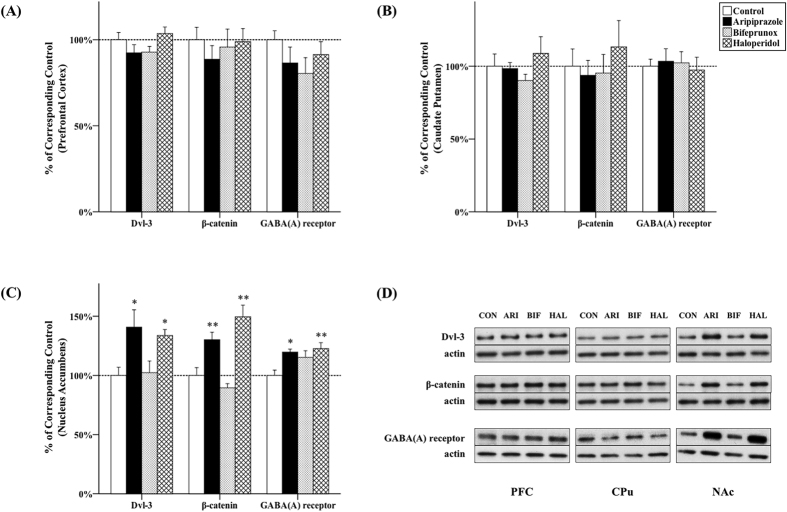
Effects of three antipsychotics on Dvl-3, β-catenin and GABA_A_ (β-1) receptor expression. The effects of aripiprazole (ARI), bifeprunox (BIF) and haloperidol (HAL) on the expression of Dvl-3 and β-catenin were measured in the prefrontal cortex (**A**), caudate putamen (**B**) and nucleus accumbens (**C**). The representative bands of Western blot are shown in (**D**). Dvl-3 was quantified at 85 kDa; β-catenin was quantified at 92 kDa; GABA_A_ (β-1) receptor was quantified at 54 kDa. The data were normalised by taking the average value of the control group as 100% and expressed as mean ± S.E.M. (*p ≤ 0.05, **p < 0.01 *vs* the control).

**Figure 4:**
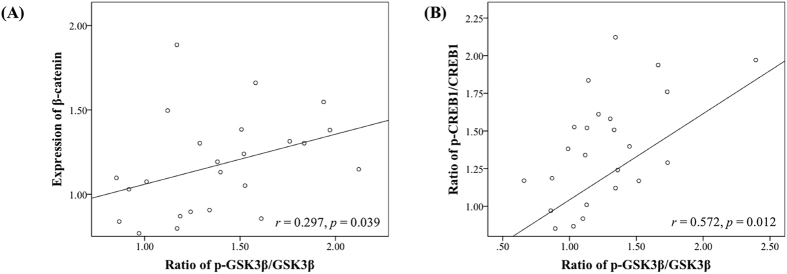
Correlations between the ratio of p-GSK3β/GSK3β and the ratio of the expression of β-catenin, and the ratio of p-CREB1/CREB1 in the NAc. The ratio of p-GSK3β/GSK3β was positively correlated with the expression of β-catenin (**A**), as well as the ratio of p-CREB1/CREB1 (**B**).

Chronic antipsychotic administration had significant effects on the expression of Dvl-3 (*F*_3, 20_ = 4.629, *p* = 0.007) and β-catenin (*F*_3, 20_ = 15.704, *p* < 0.001) in the NAc. Post-hoc tests indicated that the protein levels of Dvl-3 were significantly elevated by administration of both aripiprazole (+40.8%, *p* = 0.011) and haloperidol (+33.7%, *p* = 0.031) in the NAc; they also significantly increased the expression of β-catenin (aripiprazole, +30.3%, *p* = 0.008; haloperidol, +49.6%, *p* < 0.001) (Fig. 3C,D). Additionally, the ratio of p-GSK3β/GSK3β was positively correlated with the expression of β-catenin (*r = *0.297, *p = *0.039) (Fig. 4A). On the other hand, no significant effect was observed in the other two brain areas (Fig. 3A,B,D).

### The effect of antipsychotics on GABA_A_ receptor expression

GABA_A_ receptors containing β-1 subunit were examined in the present study. The expression of GABA_A_ β-1 receptors in the NAc was significantly affected by antipsychotic drug administration (*F*_3, 20_ = 4.926, *p* = 0.005). Both aripiprazole and haloperidol administration significantly increased GABA_A_ β-1 receptor expression in the NAc, (aripiprazole, +19.8%, *p* = 0.008; haloperidol, +22.7%, *p* = 0.003) (Fig. 3C,D) but not in the PFC and CPu (Fig. 3A,B,D).

### The effects of antipsychotics on NMDAR subunits expression

#### PFC

**Figure 5:**
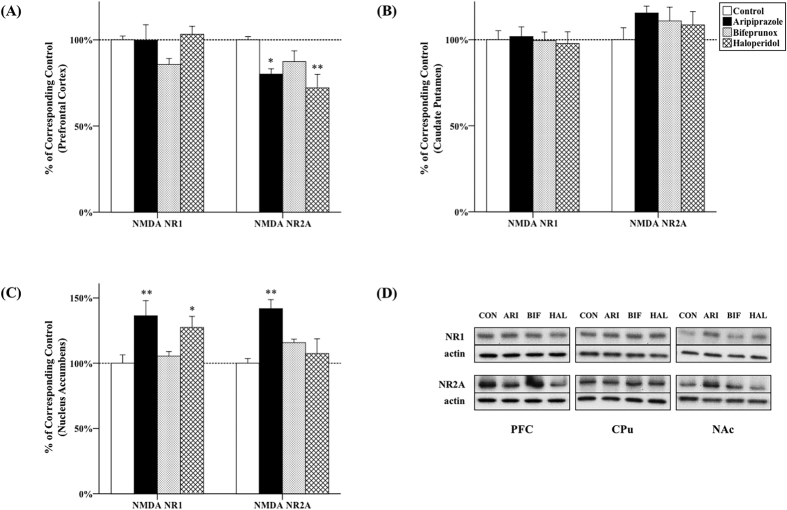
Effects of three antipsychotics on NMRA receptor subunits expression. The effects of aripiprazole (ARI), bifeprunox (BIF) and haloperidol (HAL) on the expression of NMDA receptor subunits NR1 and NR2A were measured in the prefrontal cortex (**A**), caudate putamen (**B**) and nucleus accumbens (**C**). The representative bands of Western blot are shown in (**D**). NR1 was quantified at 105 kDa; NR2A was quantified at 165 kDa. The data were normalised by taking the average value of the control group as 100% and expressed as mean ± S.E.M. (*p ≤ 0.05, **p < 0.01 *vs* the control).

The expression of NR2A was significantly altered by antipsychotic drug administration in the PFC (*F*_3, 20_ = 4.976, *p* = 0.010), but not NR1 (*F*_3, 20_ = 1.067, *p* = 0.137). Post-hoc tests revealed that NR2A levels were reduced by both aripiprazole (−19.9%, *p* = 0.020) and haloperidol (−27.9%, *p* = 0.002) (Fig. 5A,D).

#### CPu

The expression of NR1 and NR2A was not affected by drug administration in the CPu (NR1, *F*_3, 20_ = 0.082, *p* = 0.969; NR2A, *F*_3, 20_ = 0.909, *p* = 0.454) (Fig. 5B,D).

#### NAc

Both NR1 and NR2A expression was significantly changed by antipsychotic drug administration (NR1, *F*_3, 20_ = 4.653, *p* = 0.013; NR2A, *F*_3, 20_ = 6.923, *p* = 0.002). Post-hoc tests showed that aripiprazole increased the expression of both NR1 (+36.3%, *p* = 0.006) and NR2A (+41.9%, *p* = 0.001); haloperidol also elevated NR1 expression (+27.4%, *p* = 0.033) in the NAc (Fig. 5C,D).

### The effect of antipsychotics on CREB1 activity

**Figure 6:**
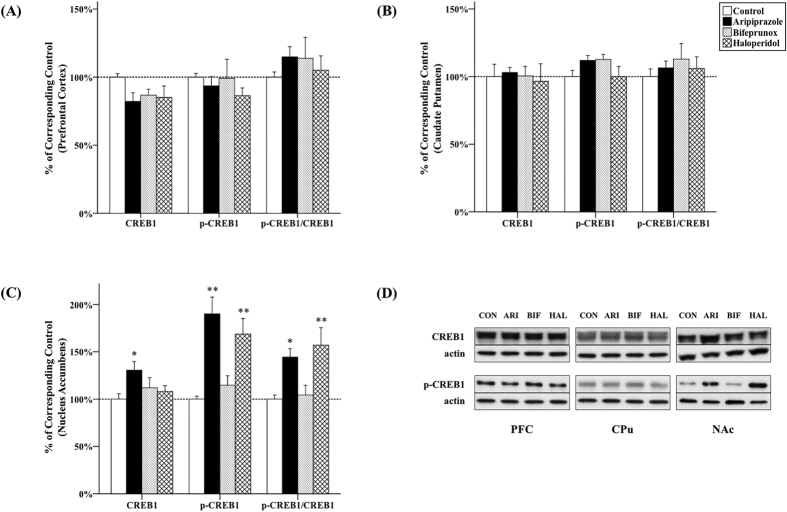
Effects of three antipsychotics on CREB1 activity. The effects of aripiprazole (ARI), bifeprunox (BIF) and haloperidol (HAL) on CREB1 activity were measured in the prefrontal cortex (**A**), caudate putamen (**B**) and nucleus accumbens (**C**). The representative bands of Western blot are shown in (**D**). CREB1 was quantified at 40 kDa; p-CREB1 was quantified at 37 kDa. The data were normalised by taking the average value of the control group as 100% and expressed as mean ± S.E.M. (*p ≤ 0.05, **p < 0.01 *vs* the control).

The levels of CREB1 and p-CREB1 in the PFC and CPu were not altered by any of the three antipsychotic drug administration (Fig. 6A,B,D). In the NAc, antipsychotic drug administration had a significant effect on CREB1 (*F*_3, 20_ = 2.502, *p* = 0.045), p-CREB1 (*F*_3, 20_ = 10.698, *p* < 0.001), as well as the ratio of p-CREB1/CREB1 (*F*_3, 20_ = 5.972, *p* = 0.002). Post-hoc tests showed that the expression of CREB1 was significantly elevated by administration of aripiprazole (+30.5%; *p* = 0.020). Additionally, the levels of p-CREB1 were significantly promoted by aripiprazole (+90.0%, *p* < 0.001) and haloperidol (+68.8%, *p* = 0.002) in the NAc (Fig. 6C,D); they also elevated the ratio of p-CREB1/CREB1 (aripiprazole, *p* = 0.019; haloperidol, *p* = 0.004) (Fig. 6C). Furthermore, the ratio of p-GSK3β/GSK3β was positively correlated with the ratio of p-CREB1/CREB1 (*r *=* *0.572, *p = *0.012) in the NAc (Fig. 4B).

### The effect of antipsychotics on PKA activity

The levels of PKA-Cα and p-PKA-C were not changed in any brain regions after chronic antipsychotic administration in the present study (data not shown).

## Discussion

The present study investigated the chronic effects of aripiprazole on Akt-GSK3β and Dvl-GSK3β-β-catenin signalling, PKA activity, GABA_A_ (containing β-1) receptor expression, NMDAR subunits expression and CREB1 activity in three brain regions, compared with haloperidol and bifeprunox. This is the first study to examine the chronic *in vivo* effects of aripiprazole on these cellular signalling pathways. The results have shown that all three antipsychotics had different effects on these signalling pathways, depending on the antipsychotic and the brain region.

Elevated Akt-GSK3β signalling in the pathophysiology of schizophrenia has been reported in a number of studies^4^,^7^,^44^,^45^,^46^,^47^. Various antipsychotics have shown increasing effects on the Akt-GSK3β signalling pathway in previous studies^7^,^8^,^9^,^48^,^49^. Previous acute and 1-week studies have also demonstrated that both aripiprazole and haloperidol increase the levels of p-GSK3β, but not p-Akt^12^,^42^. In the current study, chronic administration of all three drugs was able to increase the ratio of p-Akt/Akt and p-GSK3β/GSK3β in various brain regions, indicating reduced GSK3β activity in these brain areas; this is generally consistent with the findings of previous acute and short-term studies. It is worthy to note that aripiprazole is not only a D_2_R partial agonist, but also a biased antagonist at the β-arrestin2^50^. It has been shown that inhibition of D_2_R mediated β-arrestin2-GSK3β signalling pathway is a common property that contributes to the therapeutic effects of various antipsychotics, including aripiprazole^50^,^51^,^52^, which has been confirmed in this chronic study. It should also be noted that antipsychotics affect Akt-GSK3β signalling in a time-dependent manner. Roh *et al*.^53^ found that the duration of p-Akt signalling induced by antipsychotics (about 1 hour) was much shorter than that of p-GSK3β. The time interval between the last administration and sacrifice in the previous^12^,^42^ and the present studies was longer than 1 hour. The levels of p-Akt were undetectable in the acute and short-term studies^12^,^42^, but were observed in this chronic study, which suggests that chronic administration of antipsychotic drugs might be able to have prolonged effects on Akt activity and thereby on Akt-GSK3β signalling.

**Figure 7:**
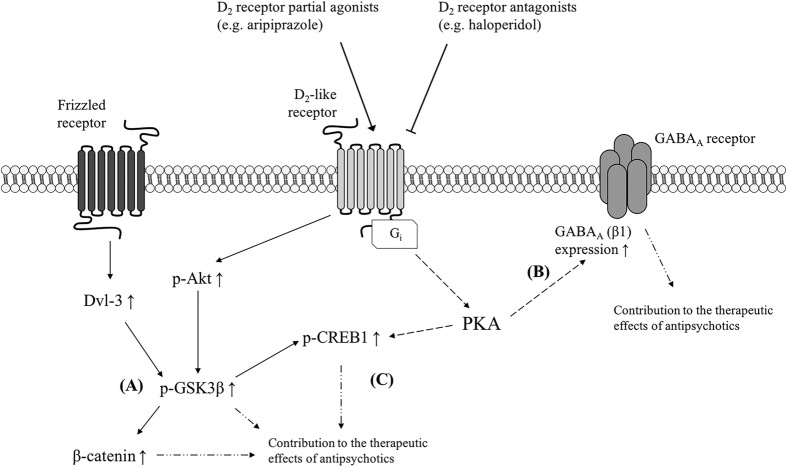
A proposed schematic diagram illustrating the chronic effects of aripiprazole and haloperidol on cellular signalling in the nucleus accumbens. D_2_ receptor antagonists (e.g. haloperidol) and D_2_ receptor partial agonists (e.g. aripiprazole) bind with the dopamine D_2_-like receptor, resulting in the phosphorylation of Akt and the subsequent phosphorylation of GSK3β probably via biasedly antagonising D_2_R-mediated β-arrestin2, together with the activation of the Dvl-GSK3β-β-catenin pathway (**A**), which might contribute to the therapeutic effect of antipsychotics. On the other hand, antagonism of the D_2_-like receptors by aripiprazole and haloperidol leads to increased expression of GABA_A_ (β-1) receptors probably via the activation of PKA activity (indicated as a dashed arrow) (**B**), and enhanced CREB1 activity via the activation of GSK3β activity and PKA activity (**C**), both of which might also be involved in the therapeutic actions of antipsychotics.

Although aripiprazole, bifeprunox and haloperidol were able to influence GSK3β activity in the present study, their effects on GSK3β were not identical in each brain region. In the PFC, administration of aripiprazole and bifeprunox, but not haloperidol, was able to suppress the activity of GSK3β via the Akt-GSK3β signalling pathway. Aripiprazole and bifeprunox also decreased total protein levels of both Akt and GSK3β in response to the chronic treatment of these two drugs. It has been suggested that inhibition of GSK3β contributes to the therapeutic effects of antipsychotics^48^ and the functional impairment in the PFC is related to the negative symptoms and cognitive deficits of schizophrenia^54^. Our results suggested that the therapeutic effects of aripiprazole on the negative symptoms and cognitive deficits of schizophrenia might be attributed to its inhibiting effects on GSK3β activity in the PFC. Furthermore, bifeprunox was able to improve the negative symptoms of schizophrenia in clinical trials^55^ and it inhibited the activity of GSK3β in the present study, which further confirms that suppression of GSK3β activity is very likely to be associated with the effects of antipsychotics on the negative and cognitive symptoms of schizophrenia. In contrast, haloperidol did not display any significant effects on GSK3β in the PFC in the present study and a previous acute study^42^, which may explain why haloperidol does not have therapeutic effects on the negative symptoms and cognitive deficits of schizophrenia. It should be noted that the present study did not found any changes of β-catenin, CREB1 and GABA_A_ receptor in the PFC. Therefore, whether antipsychotics control the negative symptoms and cognitive deficits of schizophrenia via the enhancement of Akt-GSK3β signalling requires further validations. In the NAc, administration of all three drugs increased the phosphorylation of both Akt and GSK3β in the present study. Since abnormal function of the NAc is linked to the positive symptoms of schizophrenia^56^, our results indicated that the suppression of GSK3β functions via the Akt-GSK3β signalling pathway in the NAc is very likely to be involved in the therapeutic effects of antipsychotics (probably on the positive symptoms of schizophrenia) (Fig. 7A). In the CPu, chronic treatment with these antipsychotics had very limited effects on the Akt-GSK3β signalling pathway, which suggests that GSK3β activity in the CPu may not be involved in the long-term clinical effects of antipsychotics. Yager *et al*.^57^ have reviewed that the NAc and CPu are heterogeneous structures with different neural inputs and outputs connected with various brain regions. For example, the NAc receives dopaminergic inputs from the ventral tegmental area, while sending outputs to limbic areas and the PFC; the CPu receives dopaminergic inputs from the substantia nigra pars, while sending projections to neocortical areas^57^. Therefore, chronic antipsychotic treatment might have different in the NAc and CPu. However, the brain-regional differences in the effects of antipsychotics require further clarification.

Previously, various classes of antipsychotics (e.g. aripiprazole, haloperidol, clozapine and risperidone) have been found to be able to increase the expression of Dvl-3 and/or β-catenin in various brain regions of animals, including the frontal cortex and striatum, indicating enhanced activity of β-catenin in these brain areas^8^,^9^,^10^,^11^. The current data has demonstrated that Dvl-3 and β-catenin expression was significantly increased by administration of both aripiprazole and haloperidol in the NAc only. These results are partly consistent with those of previous studies. Since dysfunction of the NAc is associated with the positive symptoms of schizophrenia and the main target of antipsychotics^56^ as mentioned above, the present study suggests that regulation of β-catenin via GSK3β might be a common mechanism by which antipsychotics exert their effects, even if they have different pharmacological profiles (Fig. 7A). It is further suggested that the increased expression of β-catenin in the NAc is very likely to be a route through which antipsychotics exert their therapeutic effects, especially on the positive symptoms of schizophrenia.

It has been reported that chronic haloperidol administration increased the binding of GABA_A_ receptors in the NAc; and both haloperidol and clozapine administration also increased GABA_A_ receptor binding in the limbic cortex^25^. A recent short-term study has demonstrated that both aripiprazole and haloperidol increased GABA_A_ (containing β-1) receptor expression in the NAc (although the increasing effect of haloperidol did not reach significance)^17^, which is validated by the present study. In this study, we found that chronic administration of aripiprazole and haloperidol were able to significantly increase GABA_A_ (containing β-1) receptor expression in the NAc, but no effects were shown in the other two brain regions. Since dysfunction of the NAc is related to the positive symptoms of schizophrenia^56^ as mentioned above, our findings together with other studies, suggest that increased GABA_A_ receptor expression and probably enhanced GABA_A_ signalling transmission in the NAc is very likely to be involved in the therapeutic effects of antipsychotics (possibly on the positive symptoms of schizophrenia) (Fig. 7B). It is worth noting that the GABA_A_ receptor is regulated by D_2_-like receptor downstream PKA signalling^58^,^59^. A 1-week study has revealed that PKA phosphorylation levels in the NAc, paralleling the expression of GABA_A_ receptors, was increased by aripiprazole and haloperidol^17^. In this chronic study, the effects of aripiprazole and haloperidol in increasing GABA_A_ receptor expression persisted, whereas the changes in PKA activity were undetectable. The reason is unknown. It is possible that the up-regulated expression of GABA_A_ receptors by short-term antipsychotic drug administration (through PKA) is an adaptive and prolonged change; even if there was no further alteration in PKA activity after chronic administration (probably due to adaptive changes in D_2_Rs), the increased GABA_A_ receptor expression could be maintained.

A variety of studies has indicated dysfunction of NMDARs in schizophrenia^5^. In the current study, both aripiprazole and haloperidol administration was able to increase the expression of NMDAR subunits in the NAc, but reduce it in the PFC. Specifically, aripiprazole increased the expression of both NR1 and NR2A in the NAc, while haloperidol increased NR1 expression; in the PFC, both aripiprazole and haloperidol decreased NR2A levels. Previously, Segnitz *et al*.^41^ have reported that NR2A mRNA levels were decreased in the PFC by aripiprazole treatment for 4 months, but not changed in the CPu; Schmitt *et al*.^40^ have also found reduced NR2A mRNA in the PFC induced by both haloperidol and clozapine treatment. Furthermore, it was found that 4-week administration with haloperidol and D_2_-like receptor antagonist – raclopride increased the levels of both protein and mRNA of NR1 in the striatum^39^. Additionally, [^3^H]-MK-801 binding in the NAc was increased by haloperidol^40^. These previous findings are consistent with those of this study that both typical and atypical antipsychotics can increase NMDARs expression in the striatum (particularly in the NAc, but not in CPu), and reduce NR2A expression in the PFC. It is worth noting that antipsychotics have very low affinity with NMDARs. Therefore, it is necessary to further reveal through which pathway(s) antipsychotics regulate NMDARs.

Previously, haloperidol was able to increase phosphorylation levels of CREB1 in the striatum and hippocampal neuron culture^28^,^29^,^30^. In addition, amisulpride, clozapine and olanzapine were also able to induce CREB1 phosphorylation in *in vitro* studies^31^,^32^,^33^. A 1-week *in vivo* study has shown that a 1-week administration of aripiprazole increased the expression of CREB1 in the NAc^17^. In the present study, chronic administration of aripiprazole increased CREB1 expression in the NAc; and both aripiprazole and haloperidol enhanced CREB1 activity via increasing the ratio of p-CREB1/CREB1. This confirmed that CREB1 is involved in the actions of antipsychotics. Moreover, extensive communication occurs between CREB1 and PKA, and the Akt-GSK3β pathway^60^. In the present study, we did not observe any change in PKA activity, nor in the correlation between PKA and CREB1 activity in the NAc (indicated as a dashed arrow in Fig. 7C). However, GSK3β activity is positively correlated with CREB1 activity. A previous study has indicated that CREB1 activity was increased by inhibition of GSK3β in cultured rat cerebral cortical neurons^33^. Amisulpride also induced the phosphorylation of CREB1 via the Akt-GSK3β pathway in SH-SY5Y cells^32^. Since patients with novel variants in the CREB1 gene experienced the positive symptoms of schizophrenia^27^, our data suggests that activation of CREB1 via the Akt-GSK3β pathway in the NAc is very likely to be associated with the therapeutic effects of aripiprazole and haloperidol on the positive symptoms of schizophrenia (Fig. 7C). It is worth noting that Mavrikaki *et al*.^34^ suggested that phosphorylation of CREB1 was increased by 3-week injection of aripiprazole in the PFC and striatum of rats, probably through NMDA receptors. The regulation of CREB1 via NMDARs has also been confirmed by other studies^61^,^62^. The present study has shown both aripiprazole and haloperidol were able to increase CREB1 phosphorylation and NMDAR expression in the NAc, simultaneously. Whether the increased expression of NMDARs (partly) contributes to the enhanced phosphorylation of CREB1 in the NAc requires further validations. Lastly, phosphorylation of histone H3S10, an epigenetic modification, has been reported to be significantly increased in schizophrenia patients^63^. Since histone H3 phosphorylation could be regulated by G protein-coupled receptor and NMDAR signalling^64^, it is important to further investigate how the modulations of antipsychotics on these signalling pathways contribute to their therapeutic effects.

In summary, the present study has demonstrated that chronic administration of aripiprazole had different effects on the Akt-GSK3β, Dvl-GSK3β-β-catenin, GABA_A_ receptor and CREB1 activity in a brain region-dependent manner. Compared to the effects of haloperidol only in the NAc and bifeprunox mainly in the PFC, aripiprazole affected these cellular signalling pathways in both the PFC and NAc, which may explain its unique clinical effects. It is also worth noting that the present and previous studies mentioned above examined the effects of antipsychotic drugs in healthy animals. It is necessary to investigate the effects of antipsychotics in the animal models for schizophrenia and other mental disorders in future studies.

## Methods

### Animals and drug administration

Male Sprague-Dawley rats (aged 8 weeks) were obtained from the Animal Resource Centre (Perth, Australia). After arrival, all rats were housed in individual cages under environmentally controlled conditions (temperature 22 °C, light cycle from 07:00AM to 07:00PM), with *ad libitum* access to water and standard laboratory chow diet. All experimental procedures were approved by the Animal Ethics Committee (Application #: AE11/02), University of Wollongong, and complied with the Australian Code of Practice for the Care and Use of Animals for Scientific Purposes (2004). All efforts were made to minimise animal distress and prevent suffering.

Before drug administration commenced, the rats were trained for self-administration of the sweet cookie dough pellets for a week. Then the rats were randomly assigned into one of the following drug treatment groups (*n* = 6/group): aripiprazole (0.75 mg/kg; Otsuka, Japan), bifeprunox (0.8 mg/kg; Otava, Ukraine), haloperidol (0.1 mg/kg; Sigma, Australia), or vehicle. Drug powder mixed with the cookie dough pellets was delivered orally 3 times per day at 07:00AM, 03:00PM and 11:00PM for 10 weeks. This drug administration method aims to mirror the human scenario of oral administration and has been well-established in our laboratory^17^,^65^,^66^. These dosages were transferred from the recommended dosages in humans based on body surface area, according to the FDA guidelines for clinical trials^67^,^68^. A 0.75 mg/kg aripiprazole, 0.8 mg/kg bifeprunox and 0.1 mg/kg haloperidol dosage in rats is equivalent to ~7.5 mg, ~8 mg and ~1 mg in humans (60 kg body weight), respectively, all of which are within the recommended clinical dosages^55^,^69^,^70^. Moreover, the dosages used in this study have been shown to be physiologically and behaviourally effective in rodents, without inducing EPS side-effects^71^,^72^,^73^,^74^. After 10-week administration, all animals were euthanised in a CO_2_-filled chamber. Brains were immediately removed and frozen in liquid nitrogen. All animals were sacrificed between 09:00AM and 11:00AM to minimise circadian-induced variation of protein expression.

### Brain dissection

The discrete brain regions were collected using a brain microdissection puncture technique, which has been well-established^17^,^42^. Specifically, based on the brain atlas^75^, three sections through the forebrain (Bregma 3.30 to 4.20 mm) were dissected for the PFC; and three sections through the striatum (Bregma 1.00 to 2.20 mm) were dissected for the NAc and CPu, respectively. Dissected tissue was kept at −80 °C for future use.

### Western blots

Frozen brain samples were homogenised in homogenising buffer (9.8 ml NP-40 cell lysis buffer (Invitrogen, #FNN0021) mixed with 100 μl Protease Inhibitor Cocktail (Sigma-Aldrich, #P8340), 100 μl β-Glycerophosphate (Sigma-Aldrich, #G9422) and 33.3 μl phenylmethylsulfonyl fluoride (Sigma-Aldrich, #P7626)). Protein concentration of each sample was measured by the *DC* Protein Assay (Bio-Rad, #500-0111). Each sample containing 10 μg of protein was denatured at 95 °C, and loaded into 4–20% Criterion^TM^ TGX^TM^ Precast Gels (Bio-rad, #5671095). The gels were run vertically in Criterion^TM^ Vertical Electrophoresis Cell (Bio-rad, #1656001) until the proteins separated, followed by the electrophoretical transfer of the proteins to a polyvinylidene difluoride membrane in Criterion^TM^ Blotter (Bio-rad, #1704071). All membranes were then blocked by 5% skim milk powder and incubated in primary antibodies. Amersham Hyperfilm ECL (GE Healthcare, #28-9068-36) and Luminata Classico Western HRP substrate (Millipore, #WBLUC0500) were used to visualise the immunoreactive bands. All Western blot experiments were performed in duplicate to ensure consistency.

The antibodies used in the present study to examine PKA activity were anti-PKA-Cα (1:1000; Santa Cruz, #SC-903) and anti-phosphor-PKA-C (Thr197) (1:1000; Cell Signalling, #5661). The antibodies used to examine the GSK3β-involved pathways were anti-Akt (1:2000; Cell Signalling, #4691), anti-phosphor-Akt (Thr308) (1:1000; Cell Signalling, #13038), anti-GSK3β (1:2000; Cell Signalling, #5676), anti-phospho-GSK3β (Ser9) (1:1000; Cell Signalling, #9322), anti-Dvl-3 (1:1000; Santa Cruz Biotechnology, #SC-8027), anti-β-catenin (1:1000; Santa Cruz Biotechnology, #SC-7963). The antibodies used to detect subunits of GABA_A_ receptors were: anti-GABA_A_ β-1 (1:1000; Abcam, #ab154822), anti-GABA_A_ β-2 (1:1000; Abcam, #ab156000), and anti-GABA_A_ β-3 (1:1000; Abcam, #ab98968). The antibodies used to examine NMDAR were anti-NMDAR1 (1:2000, Abcam, #ab109182) and anti-NMDAR2A (1:500, Abcam, #ab124913). In addition, anti-CREB1 (1:2000, Abcam, #ab32515) and anti-phospho-CREB11 (1:2000, Abcam, #ab32096) were used to measure the activity of CREB1. Mouse anti-actin primary polyclonal antibody (1:10000; Millipore, #MAB1501) was used to determine the actin levels. The secondary antibodies were HRP-conjugated anti-rabbit IgG antibody (1:3000; Cell Signalling, #7074) and HRP-conjugated anti-mouse IgG antibody (1:3000; Cell Signalling, #7076). Examples of full pictures of the blots for each antibody are shown in the Supplementary file.

### Statistics

All data were analysed using SPSS Statistics V22.0 program. The immunoreactive signals were quantified using Bio-Rad Quantity One software. The data of each targeted protein were then corrected based on their corresponding actin levels. Data normal distribution was tested using histograms and a Kolmogorov-Smirnov *Z* test. For statistical evaluation, one-way analysis of variance (ANOVA) was performed if the data was normally distributed. The post-hoc Dunnett *t* test was used to compare each drug treatment group with the control group. The results were normalised by taking the value of the control group as 100%. Pearson’s correlation test was used to analyse the relationships among the measurements. Statistical significance was accepted when *p* ≤ 0.05.

## Additional Information

**How to cite this article**: Pan, B. *et al*. Chronic administration of aripiprazole activates GSK3β-dependent signalling pathways, and up-regulates GABA_A_ receptor expression and CREB1 activity in rats. *Sci. Rep.* **6**, 30040; doi: 10.1038/srep30040 (2016).

## Supplementary Material

Supplementary Information
