# Supplementary material for: Chronic administration of aripiprazole activates GSK3β-dependent signalling pathways, and up-regulates GABAA receptor expression and CREB1 activity in rats
Source: Sci Rep. 2016 Jul 20;6:30040. doi: 10.1038/srep30040 (PMC4951756; doi:10.1038/srep30040)
Supplement: Supplementary Information [file srep30040-s1.pdf]

Chronic administration of aripiprazole activates GSK3β-dependent signalling pathways, and up-regulates $GABA_A$ receptor expression and CREB1 activity in rats

Authors: Bo Pan[1,2], Xu-Feng Huang[2], Chao Deng[1,2 *]

1: Antipsychotic Research Laboratory, Illawarra Health and Medical Research Institute, Wollongong, 2522, NSW, Australia

2: Centre for Translational Neuroscience, School of Medicine, University of Wollongong, Wollongong, 2522, NSW, Australia

*Corresponding Author:
Prof. Chao Deng, Illawarra Health and Medical Research Institute, University of Wollongong, Wollongong, 2522, NSW, Australia
E-mail: chao@uow.edu.au, Tel: (+61 2) 4221 4934, Fax: (+61 2) 4221 8130

Fig. 1
Akt

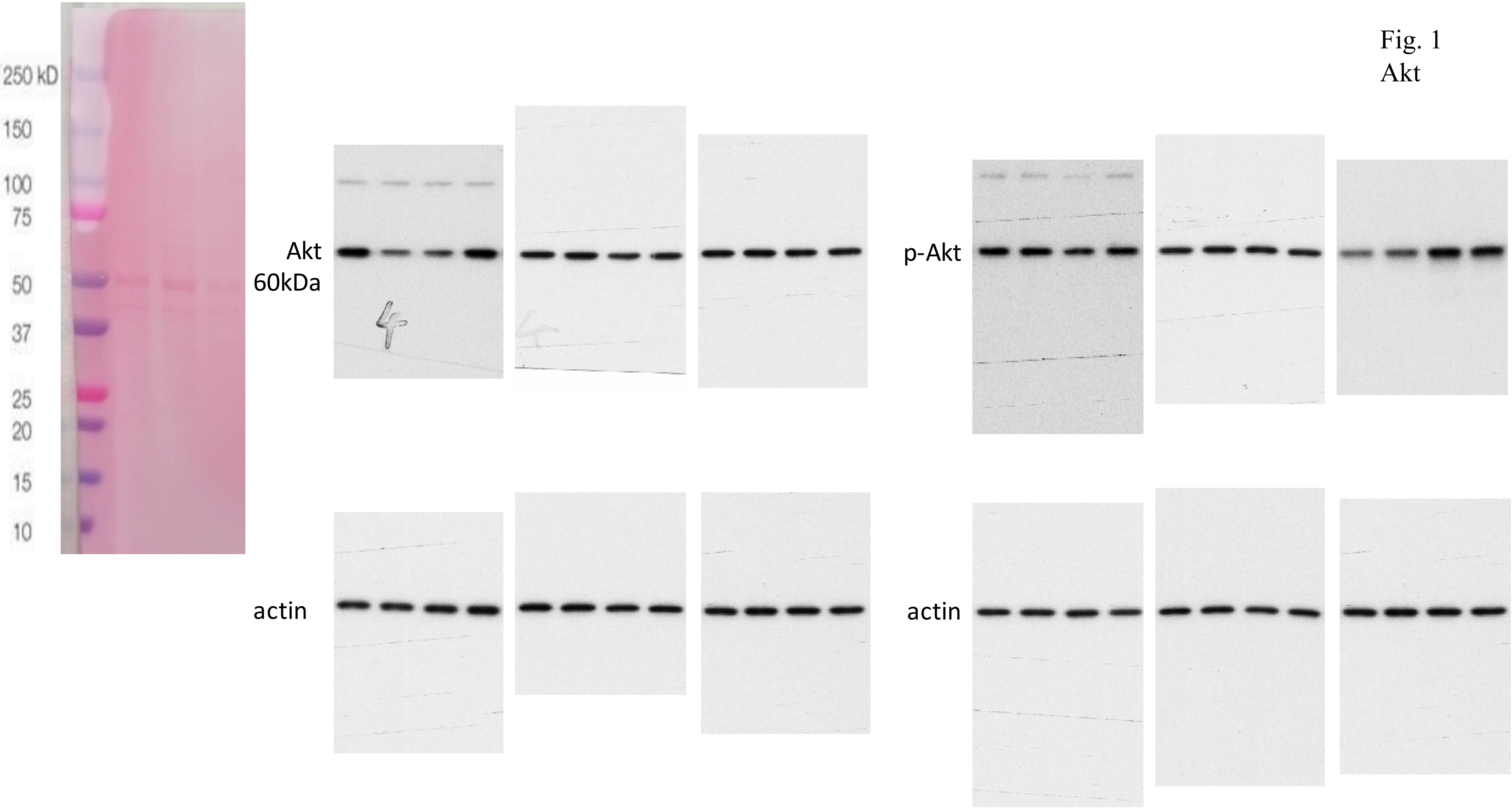

Fig. 2
GSK3β

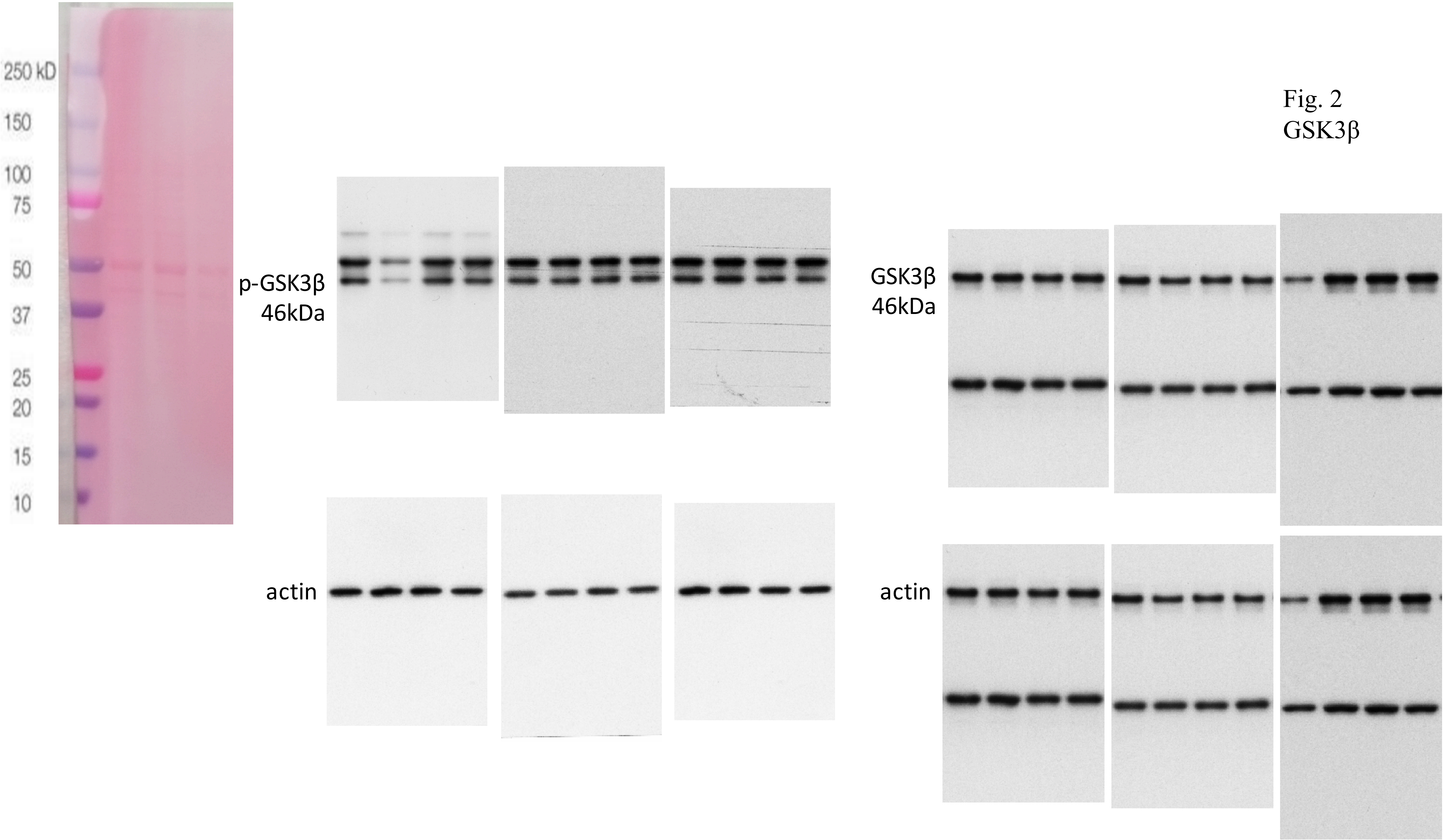
250 kD
150
100
75
50
37
25
20
15
10
p-GSK3β
46kDa
actin
GSK3β
46kDa
actin

Fig. 3
Dvl β-catenin

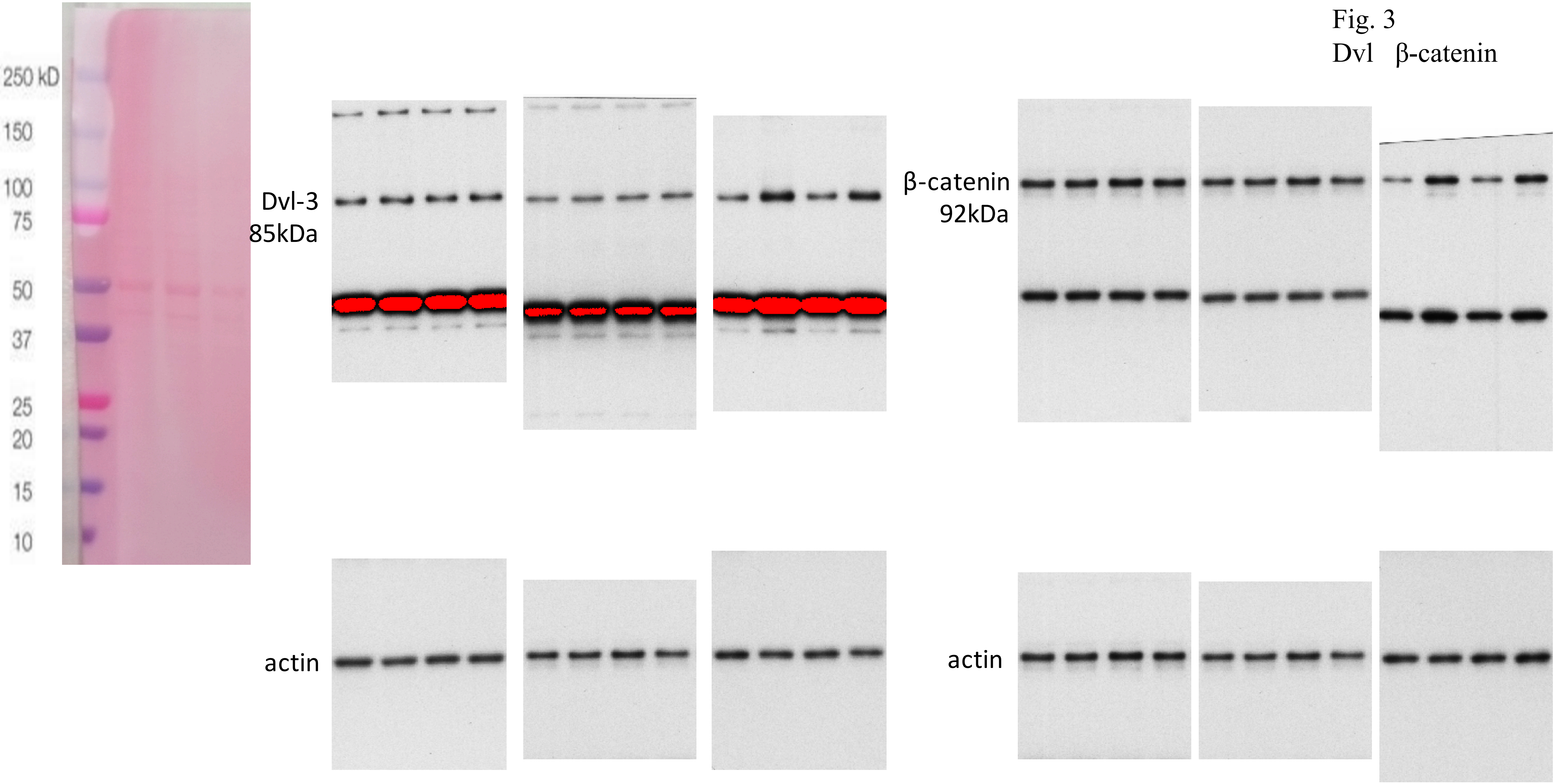

Fig. 3
GABA$_A$ receptor

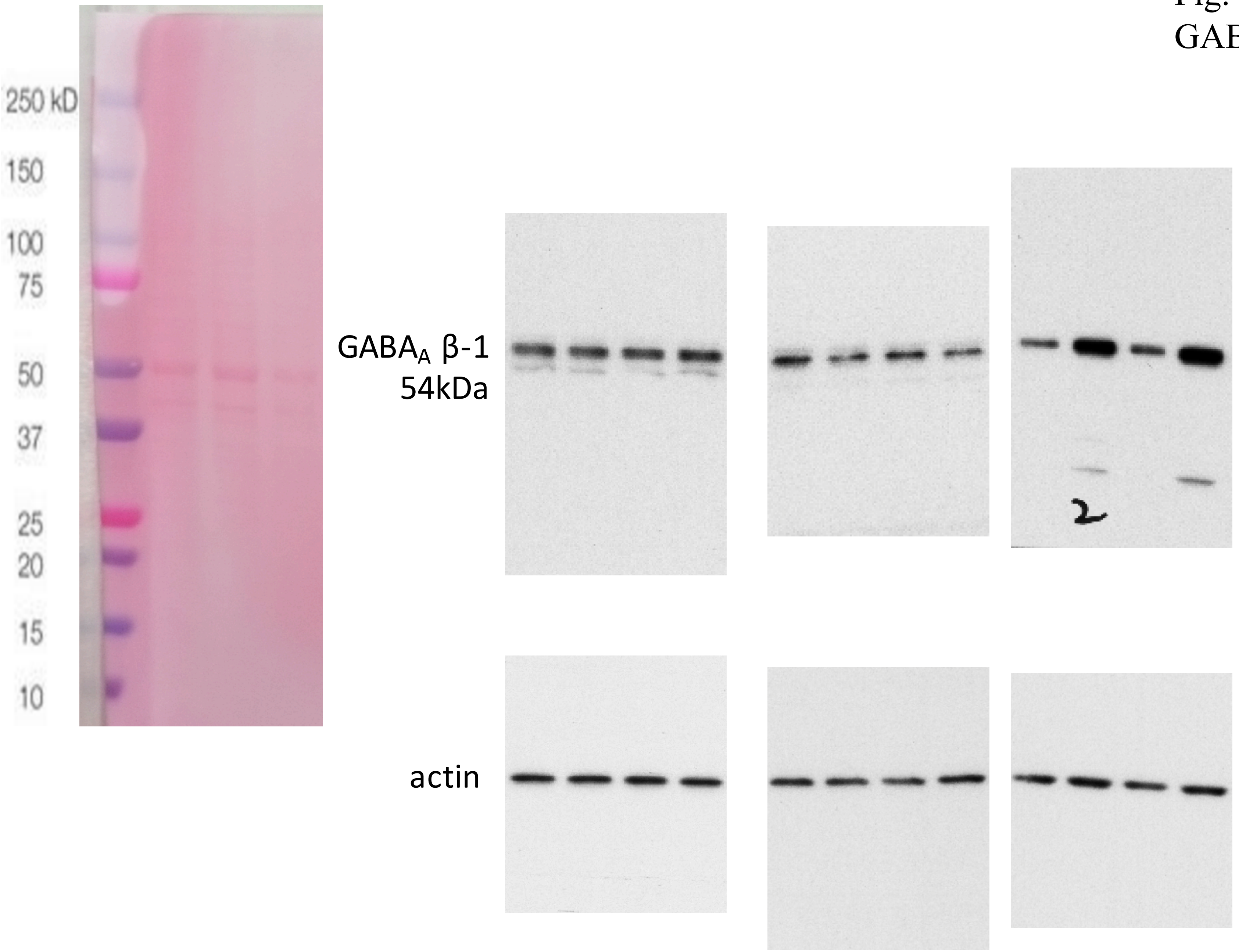

Fig. 5
NMDAR

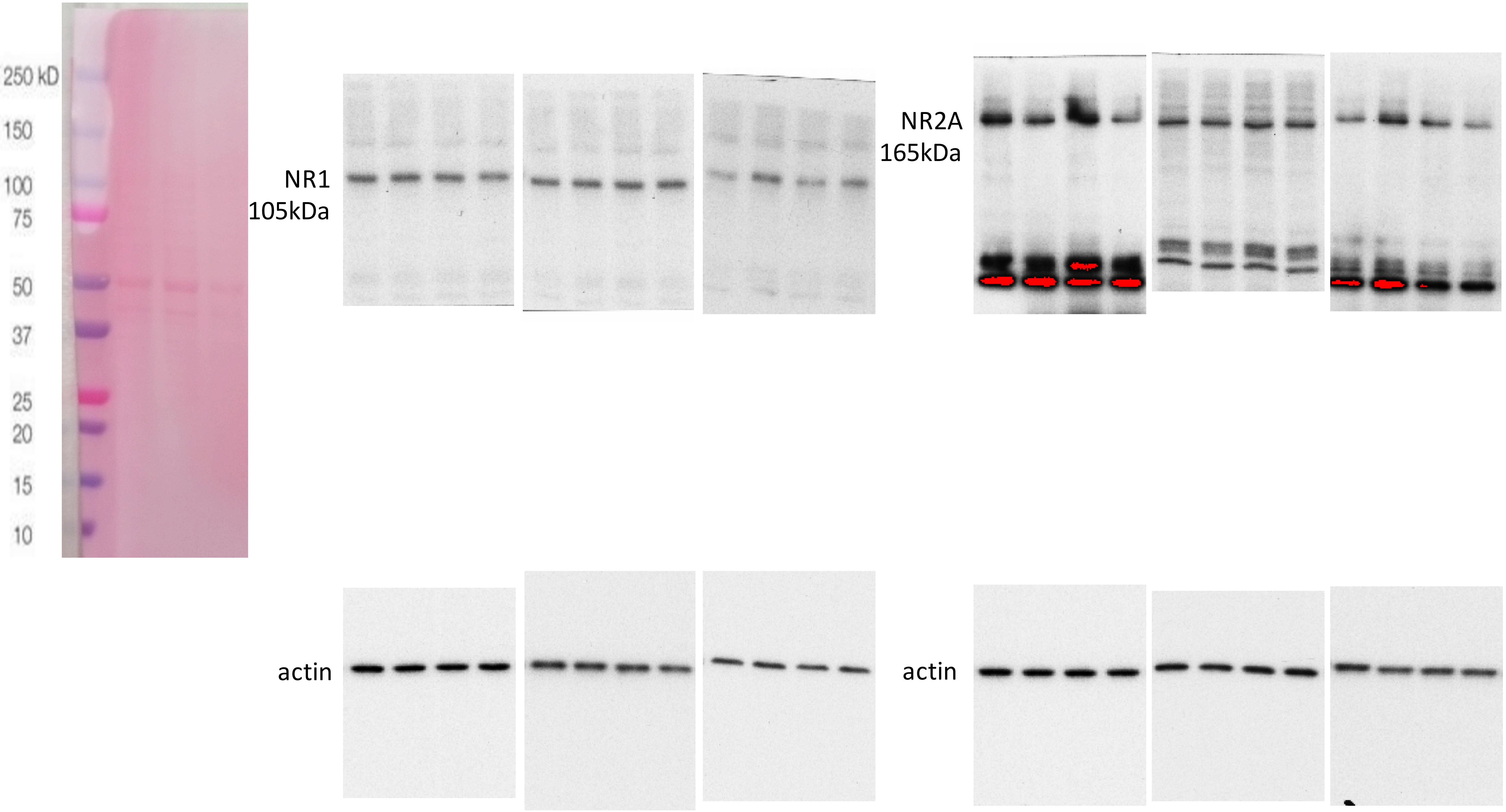

250 kD
150
100
75
50
37
25
20
15
10
NR1
105kDa
NR2A
165kDa
actin
actin

Fig. 6
CREB1

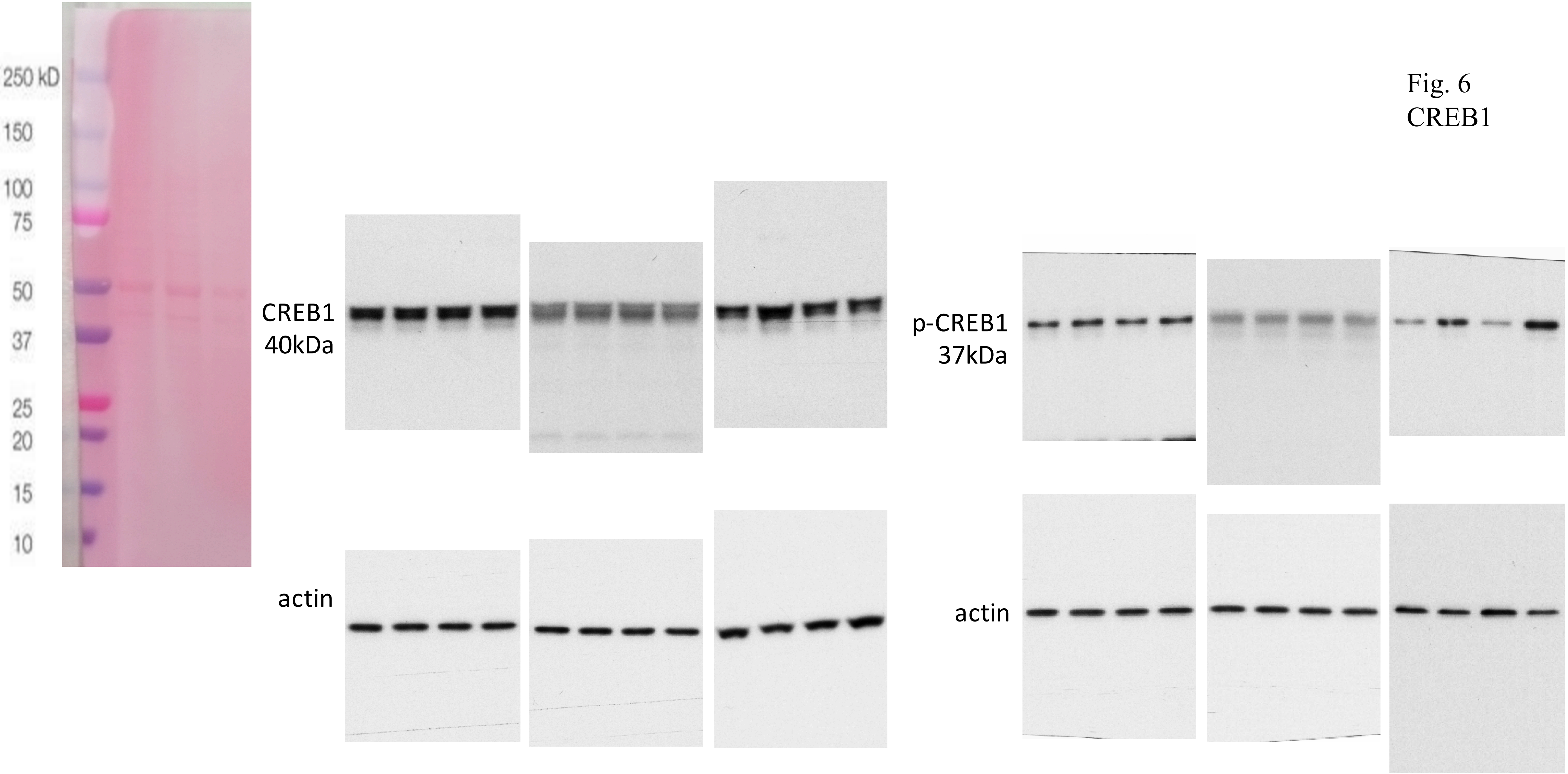
250 kD
150
100
75
50
37
25
20
15
10
CREB1
40kDa
actin
p-CREB1
37kDa
actin
